# An interaction-dominant perspective on reading fluency and dyslexia

**DOI:** 10.1007/s11881-012-0067-3

**Published:** 2012-03-30

**Authors:** M. L. Wijnants, F. Hasselman, R. F. A. Cox, A. M. T. Bosman, G. Van Orden

**Affiliations:** 1 Behavioural Science Institute, Radboud University Nijmegen, P.O. Box 9104, 6500 Nijmegen, the Netherlands; 2 Department of Developmental Psychology, University of Groningen, Groningen, the Netherlands; 3 CAP Center for Cognition, Action and Perception, University of Cincinnati, Cincinnati, OH USA

**Keywords:** Developmental dyslexia, Reading fluency, Recurrence quantification analysis, Self-organization, 1/*f* noise

## Abstract

The background noise of response times is often overlooked in scientific inquiries of cognitive performances. However, it is becoming widely acknowledged in psychology, medicine, physiology, physics, and beyond that temporal patterns of variability constitute a rich source of information. Here, we introduce two complexity measures (1/*f* scaling and recurrence quantification analysis) that employ background noise as metrics of reading fluency. These measures gauge the extent of interdependence across, rather than within, cognitive components. In this study, we investigated dyslexic and non-dyslexic word-naming performance in beginning readers and observed that these complexity metrics differentiate reliably between dyslexic and average response times and correlate strongly with the severity of the reading impairment. The direction of change in the introduced metrics suggests that developmental dyslexia resides from dynamical instabilities in the coordination among the many components necessary to read, which could explain why dyslexic readers score below average on so many distinct tasks and modalities.

When learning to read, young children must develop stable, yet flexible, relations among graphemes and phonemes. Reading fluently means coordinating these often inconsistent relations with the perceptual and motor processes necessary to read. A failure to achieve such flexible stability or coordination thus results in a failure to read fluently (see Bosman, Vonk, & van Zwam, 2006). For instance, developmental dyslexia results in slow and/or inaccurate reading performance. But the possible cause of developmental dyslexia, however, is still much debated after decades of intensive research.

One factor that troubles the search for single causes of dyslexia is the long list of criteria that is held to differentiate among dyslexic and average readers. For instance, dyslexic readers have been found to score below average on perceptual, motor and cognitive skills pertaining to speech and language, working memory, attention, ordering and sequencing, temporal processing, balance and motor control, auditory and tactile processing, mental calculations, and much more (e.g., Elliott & Gibbs, 2008). Moreover, it appears that neither of those criteria by themselves is essential for diagnosis nor specific to developmental dyslexia (e.g., Ramus, 2003). In fact, neither phonological awareness nor biological factors alone can provide a full account for the plethora of empirical findings (e.g., Blomert & Willems, 2010; Snowling, 2008; Torgesen, 2007). And in neuroscience, equally, a bewildering range of anatomical differences is held to differentiate between children with developmental dyslexia and average readers. These include reductions in temporal lobe, frontal lobe, caudate, thalamus and cerebellum (Brown et al., 2001), insula, anterior superior neocortex, posterior cortex (Pennington, 1999), occipital cortex (Eckert et al., 2003), and relative increases in the size of temporal and parietal plana (Green et al., 1999).

The observation, that so many different processes or components may contribute to the learning disability, constitutes an interesting observation in itself which poses specific challenges to any theory of developmental dyslexia (Démonet, Taylor, & Chaix, 2004; Hasselman, 2012, pp. 29–31; Ramus, 2003). Dealing with this plentitude of possibly deficient components is not trivial, especially since many effects appear to be extremely context specific from time to time (Blomert, & Mitterer, 2004; Holden, Choi, Amazeen, & Van Orden, 2011; Manis, & Keating, 2005; Ramus & Szenkovits, 2008; Van Orden, Holden, Podgornik, & Aitchison, 1999). If one additionally considers the variety of brain regions implicated in dyslexia (e.g., Leonard, Eckert, Given, Virginia, & Eden, 2006), it becomes even more difficult, if not impossible, to pinpoint a single deficient region or component of the brain whose malfunctioning uniquely leads to developmental dyslexia. Therefore, some authors have questioned whether there is in fact one isolable mechanism, deficient in dyslexic reading, which specifically serves the function of decoding written language (Bosman & de Groot, 1996; Elliott & Gibbs, 2008; Van Orden, Pennington, & Stone, 2001).

The idea, that there may not be an isolable causal source of developmental dyslexia, may not even be as strange as it appears. For one, the task of becoming literate is undoubtedly complex, irregular, and subservient to other linguistic and cognitive abilities and, therefore arguably a multifaceted process (Wallot & Van Orden, 2011a). As an example, learning to read is essentially multi-sensory in nature (Blomert, 2011; Lankhorst, Bosman & Didden, 2008). In addition, around 70 muscles must coordinate to pronounce a single utterance (Turvey, 2007). Successful reading may therefore emerge from a multitude of interdependent processes (e.g., Holden, Van Orden, & Turvey, 2009; Kello & Van Orden, 2009). In fact, in fluent reading the intrinsic dynamics of the components themselves may matter less than the mutual interdependence among those components (Van Orden & Holden, 2002; Rueckl, 2002; Van Orden & Kloos, 2003).

Nonetheless, experimental designs generally aim at comparing the measured variables as treatment cells to expose single, causally potent, sources of variance, as in an ANOVA (i.e., simple cause-and-effect relations). This means that many studies are exposed to infer the workings of the independent components and subcomponents of sensation, perception, reading, and articulation, each representing independent cognitive functions. These components are usually assumed to concatenate their effects like a row of time-ordered falling dominos, each affecting the next in its turn, often spanning several levels of analysis from the biological to the cognitive and behavioral domain (cf. Ramus, 2004). Although the merits of this approach are well acknowledged, this approach in isolation is limited nonetheless by the inability to reveal structure not contemplated by ANOVAs or other variance component designs (see Gilden, 2001; Van Orden, Holden, & Turvey, 2003).

Specifically, experiments designed to reveal independent processing components assign background noise the status of unexplained variance. That is, it is assumed that response series fluctuate randomly around a more or less constant mean. The underlying assumption then is that slower response times indicate a defect in one of the discrete processing operations that additively determine the duration of the response process. However, it is now becoming clear that trial-by-trial variability rarely constitutes white, Gaussian noise. Rather, complex temporal dependencies carry over timescales up to minutes of performance (Gilden, 2001; Riley & Turvey, 2002; Riley & Van Orden, 2005; Van Orden et al., 2003). With this in mind, the present study treats response variability as a trial-ordered response series to evaluate reading performance, and unconventionally, does not concern isolated components of cognitive architecture. The question posed is rather how the essential cognitive activities interact, and become so entangled, to give rise to fluent reading.

With this question in mind, we employed two complexity measures (cf. Wallot & Van Orden, 2011b) to investigate the temporal structure of response variability of dyslexic and non-dyslexic word-naming performance. These metrics, known as 1/*f* scaling and recurrence quantification analysis (RQA), were used to provide a characterization of the dynamical dependencies among the ongoing processes involved in dyslexic and non-dyslexic reading performance. First, we introduce these metrics, and then, we formulate our hypothesis about the nature of developmental dyslexia, based on these metrics.

## 1/*f* scaling

1/*f* ^*α*^ scaling presents a phenomenon that is receiving growing interest in psychology and beyond. It is a describing property of the trial-by-trial variability of a time series and is observed most clearly during repeated human performances when faced with the same task in stable conditions. 1/*f* scaling indicates that the magnitude of variation in response latencies is proportional to the timescale on which it is measured, thus composing a complex sequence effect spanning over the entire time course of an experiment. For a response series which composes random noise, it is fair to assume that cognitive operations are initiated by the stimulus and finish together with the response. As the extent of 1/*f* scaling increases (and hence, departures from randomness), however, this signifies the presence of processes that extend beyond the time boundaries of single trials and interact across interdependent timescales of performance.

**Fig. 1:**
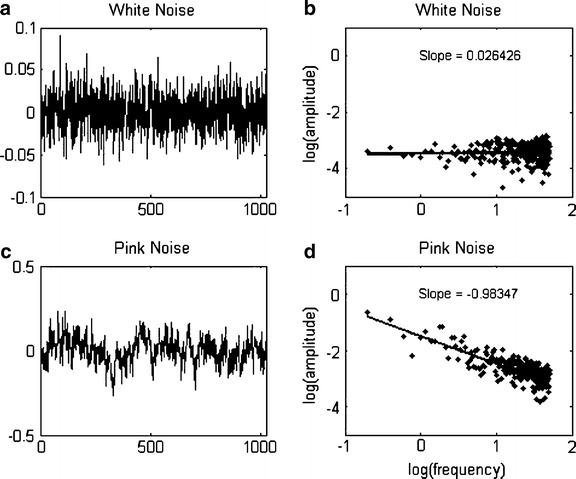
**a**–**d** Typical examples of white noise (**a**) and 1/*f* scaling (**c**) and their respective power spectra (**b**, **d**)

Specifically, 1/*f* scaling of a response time series means that changes in power (the amplitude of changes in response latency over trials) are typically small at the highest frequencies in the time series (i.e., extending over few trials), but that those changes are embedded in overarching, lower frequent changes of higher amplitude (spanning over a larger scale of measurement). 1/*f* scaling thus composes a nested pattern of response variability across scales. This can be seen in a time series plot of 1/*f* fluctuations (i.e., Fig. 1c), the series has the same “look and feel” as one zooms in or out to see more fine-grained or coarse-grained features of the fluctuations. It follows that a 1/*f* scaling relation can be expressed as a relation between the size of changes (power) and how often changes of that size occur (frequency), which is inversely proportional on logarithmic scales, see Fig. 1d. In contrast, a time series consisting of independent repeated measurements (i.e., white, random, Gaussian noise) does not represent such a relationship (see Fig. 1a, b).

Time-evolutionary properties like 1/*f* scaling are essential, because they are not visible, and even discarded, in most standard statistical analyses of cognitive performance (Riley & Turvey, 2002; Slifkin & Newell, 1998) while they do effectively distinguish between experimental conditions (e.g., Chen, Ding, & Kelso, 1997; Kello, Beltz, Holden, & Van Orden, 2007; Gilden & Hancock, 2007; Wijnants, Bosman, Hasselman, Cox, & Van Orden, 2009). In fact, structured variability (i.e., 1/*f* scaling) appears to be the rule rather than the exception in cognitive performances and is often as revealing as aggregate information in terms of unpacking the nature of the system organization (e.g., Ihlen & Vereijken, 2010; Hausdorff, 2007; Kello et al., 2007; Van Orden et al., 2003). To date, dozens of studies have been published on long-range dependence in cognitive and motor performance, all demonstrating widespread 1/*f* scaling (e.g., Kello et al., 2007; Van Orden, Kloos, & Wallot, 2011, are reviews). But although 1/*f* scaling has been observed throughout human physiology and behavior in varying degrees, its origin and meaning remain unclear (Diniz et al., 2011; Van Orden et al., 2003, 2005; Wagenmakers, Farrell, & Ratcliff, 2005).

One position in the ongoing debate is that 1/*f* scaling is a typical behavior of self-organizing systems, which reflects a fundamental aspect of all physiological and cognitive functions, which is their emergence in the balance of independent versus interdependent component activities. And in recent years, there has been a growing empirical support for the position that 1/*f* scaling indeed reflects the interaction of many ongoing processes over a multiplicity of interdependent scales, thereby serving as a coordinative basis of cognitive function (e.g., Kello et al., 2007; Van Orden et al., 2011; Wijnants et al., 2009). That is, 1/*f* scaling is usually seen most clearly in well-coordinated behaviors, and less clearly in non-optimal performance or with aging and disease (e.g., Goldberger et al., 2002; West, 2006).

For instance, deviations from 1*/f* scaling (either towards white noise or towards Brownian noise) have been found with epilepsy (Ramon, Holmes, Freeman, McElroy, & Rezvanian, 2008), heart failure (Goldberger et al., 2002), fetal distress syndrome (Goldberger, 1996), major-depressive disorder (Linkenkaer-Hansen et al., 2005), mania (Bahrami, Seyedsadjadi, Babadi, & Noroozian, 2005), attention-deficit-hyperactivity-disorder (Gilden & Hancock, 2007), autism (Lai et al., 2010), Alzheimer’s disease (Abásolo, Hornero, Gómez, García, & López, 2006), Huntington’s disease (West, 2006), Parkinson’s disease (Hausdorff, 2007), and even slow transit constipation (Yan, Yan, Zhang, & Wang, 2008). In each of these studies, healthy controls revealed long-range dynamics reliably closer to 1/*f* scaling.

In addition, the presence of 1/*f* scaling increases with learning (Wijnants et al., 2009) and decreases as task demands increase (Clayton & Frey, 1997; Correll, 2008). The presence of 1/*f* scaling also correlates, for instance, with the severity of depression symptoms (Linkenkaer-Hansen et al., 2005), the success rate of recovery from traumatic brain injury (Burr, Kirkness, & Mitchell, 2008), and falling risk in elderly (Hausdorff, 2007). In each case, more flexibly stable, adaptive, or coordinated behaviors showed clearer 1/*f* scaling. These many studies together raise the suggestion of close linkages between fractal, 1/*f* scaling dynamics and coordination in human physiology and cognition. With these precedent studies in mind, it is not unlikely that research on learning disabilities like dyslexia may benefit from an assessment of the dynamics underlying impaired reading performance.

## Recurrence quantification analysis of response times

RQA is a nonlinear technique to quantify recurring patterns and parameters pertaining to the stability and dimensionality of the underlying dynamics from a time series. Like physicists, physiologists, chemists, biologists, seismologists, physicians, economists, and more, behavioral scientists are usually confronted with systems characterized by a large number of participating, often interacting variables. RQA allows a direct access to such systems by reconstructing, from a single measured variable in the interactive system, a behavior space (or phase space) that represents the dynamics of the entire system. This reconstruction is achieved by the method of delay embedding that is based on Takens’ theorem (Takens, 1981). The phase space reconstructed from the time series of this single variable informs about the behavior of the entire system because the influence of any interdependent, dynamical variable is contained in the measured signal. The reconstruction itself involves creating time-delayed copies of the time series of a variable that become the surrogate dimensions of a multi-dimensional phase space. Consequently, the original variable becomes a dimension of the system in question and each time-delayed copy becomes another dimension of the system (Marwan, Romano, Thiel, & Kurths, 2007; Riley & Van Orden, 2005, are tutorials).

**Fig. 2:**
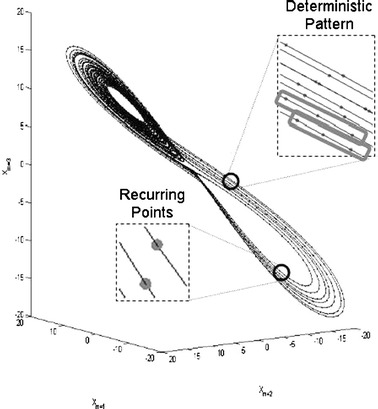
A phase space reconstruction of a highly deterministic system. The *insets* represent examples of recurrence (points that share common locations in phase space) and determinism (patterns of recurring points)

The trajectories in this multi-dimensional phase space represent the system dynamics, and the ensemble of these trajectories is called an attractor, as depicted in Fig. 2. RQA quantifies, for instance, whether a data point recurs at another point in time, or whether a sequence of recurring data points forms a recurring pattern of (multiple) neighboring data points in the reconstructed phase space. The parameters estimated by RQA include *recurrence rate* (how many data points recur or revisit shared locations in phase space, given a certain radius or neighborhood size. Recurrence rate indicates the degree of randomness in the time series or the confinement of the attractor), *determinism* (the portion of recurrent measurement values which are parts of a recurring pattern in phase space), the Shannon *entropy* of the distribution of the lengths of deterministic patterns (entropy indicates the complexity of the attractor), and *meanline* (the average length of deterministic patterns, indicative of the stability of the system). In the “Method” section, it is explained how each of these measures are computed.

To exemplify what RQA outcomes indicate exactly, consider an attractor reconstructed from a process with a steady mean imposed by random background noise, as typically assumed in reading research. The resulting (high dimensional) reconstructed phase space would yield little recurrence (neighboring points in phase space), and little or no determinism (recurring patterns of data points) because at most a few of the incidental recurrences would carry over more than one trial. The entropy measure, in RQA, indicates how much “disorder” is there in the duration of recurrent sequences. For a random signal, however, there are little if any differences (very ordered) in the duration of recurrent sequences, which typically all are very short. Therefore a random signal will carry low entropy in the distribution of the durations. Also the value of meanline is small, because the probability of observing a recurrent pattern of a given length in a stochastic signal decreases for each increase in duration. A process consisting of many intertwingled variables, on the other hand, contains a much richer dynamical history. That is, recurrence rate and determinism increase the more a system’s dynamics are dominated by interaction-dominant dynamics. Also, the entropy in the distribution of the duration of deterministic patters is higher in more complex dynamics, because more recurrent patterns (determinism) of shorter and longer duration (meanline) are observed than is ordinarily to be expected.

Over a thousand studies across scientific disciplines have used RQA to study a wide range of complex phenomena (http://www.recurrence-plot.tk/bibliography.php), including neuronal spike trains (Kaluzny & Tarnecki, 1993), breathing rhythms (Webber & Zbilut, 1994), cardiology (Marwan, Wessel, Meyerfeldt, Schirdewan, & Kurths, 2002), protein sequences (Giuliani, Benigni, Sirabella, Zbilut, & Colosimo, 2000; Manetti, Ceruso, Giuliani, Webber, & Zbilut, 1999), electroencephalographic activity (Acharya, Faust, Kannathal, Chua, & Laxminarayan, 2005; Marwan & Meinke, 2004; Thomasson, Hoeppner, Webber, & Zbilut, 2001), and electromyographic data (Webber, Schmidt, & Walsh, 1995), among other examples. As a statistical method, RQA has thus proven its worth conceptually and mathematically, to reliably and sensitively investigate complex temporal dependencies in systems that contain many interdependent variables, and consequently, emit complex and nonlinear temporal patterns of variation.

## Hypothesis

We compared the word-naming performance of young dyslexic (ages 7 to 8) and non-dyslexic (ages 6 to 7) readers. The young age of these children together with the task of naming unrelated words offered the advantage of investigating naming fluency at an early stage of reading development. This allowed us to inquire early signs of developmental dyslexia in the temporal structure of naming latencies, using 1/*f* scaling measures and RQA. As a control group we used the closest possible reading-age match to the dyslexic group.

If successful reading indeed requires multiplicative interactions among cognitive processes, we may expect that in dyslexic word-naming performance the cognitive processes necessary to read show a reduction in the extent of their mutual interactions (cf. Holden, 2002; Holden et al., 2009; Wallot & Van Orden, 2011b). This prediction aligns with the many recent findings across human physiology and cognition that reveal less clear 1/*f* scaling in less coordinated, less fluent processes. Well-coordinated behavior at the other hand possibly emerges from principles akin to self-organization. That is, a system may self-control its behavior so that it becomes governed by low dimensional dynamics that concisely meet the specific task demands at hand.

This entails for instance that, while conventional perspectives presume that different classes of words may require different mechanisms each revealed by distinct time courses (e.g., Coltheart, 1978; Coltheart, Curtis, Atkins, & Haller, 1993), a perspective of interaction-dominant dynamics allows that all response times gauge the selfsame process. Our aim was to investigate whether a more general lack of coordination among processes underlies dyslexic reading performance than would be suggested if the reading impairment were caused by a single deficient component part of the system.

With this goal in mind, 1/*f* scaling and RQA allowed measuring the relative degree of interdependence among system components. Specifically, we expected dyslexic word-naming latencies to reveal more random, higher-dimensional, less structured, and less complex dynamical signatures, relative to non-dyslexic reading performance. That is, we expected less clear examples of 1/*f* scaling in the response variability of dyslexic readers, and an underlying attractor that is less recurrent and deterministic, and yields lower entropy and meanline, compared with the response variability of average readers.

In addition, performance measures relying on 1/*f* scaling and RQA were expected to indicate the severity of a reading impairment. We exploited the inter-individual variability by correlating each of the reading performance measures to evaluate this postulate. These measures included mean response time and standardized reading scores at the one hand, and 1/*f* scaling and RQA statistics at the other. This approach allowed for an additional evaluation of the predicted relation between reading fluency and the introduced dynamical performance metrics.

## Method

### Participants

We performed a word-naming study to test the reading performance of 15 dyslexic children, ages 7 to 8. Dyslexic children were recruited in a remediation institute for dyslexia. As a control group, 15 non-dyslexic readers ranging in age from 6 to 7 years old with no history of dyslexia were recruited in a regular elementary school and performed the same task. All participants were native Dutch speakers.

## Materials

In addition to the word-naming experiment, a test known as the Een-Minuut-Test (One-Minute Test (EMT)) by Brus and Voeten (1973) was used to assess reading decoding or reading fluency. Oral reading fluency is regarded as the sole best indicator of reading problems or dyslexia (Fuchs, Fuchs, Hosp, & Jenkins, 2001). The word-naming experiment itself consisted of 550 Dutch one-syllable words (two to eight letters), with a frequency larger than 0 per million, retrieved from CELEX. Each participant was presented with one out of three randomized lists of the 550 word stimuli on a computer screen, and a voice key recorded the response time with millisecond precision.

### Procedure

All participants were orally instructed to respond as fast and as accurately as possible to the visually presented words. Then, the children were presented with 20 practice trials to make sure they understood the instruction and to calibrate the voice key. Each trial began with the presentation of a fixation point (“+++”), followed by a word. The word disappeared from the screen when an utterance was detected. The inter-stimulus interval was 500 ms for both groups of participants. After 550 stimuli were presented, the end of the experiment was announced on the screen. The reaction time series of both groups were taken to compute each participant’s average reaction time and standard deviation, as well as 1/*f* scaling and RQA measures. To the latter goal, we preceded as follows.

Before being subjected to further analyses, erroneous reaction times were removed. Erroneous reaction times either indicated that the voice key recorded a sound before the stimulus word was read, or that the voice key did not detect the pronunciation. With these largest deviations removed, reaction times larger and smaller than 2* the remaining SD from the mean were deleted. This was necessary to eliminate inherent biases in the applied time series analyses (see Holden, 2005). Then, the time series were normalized and linearly detrended. Zeros were appended to the normalized time series if a length of 512 data points required for the fractal analyses was not reached (cf. Van Orden et al., 2003). For time series longer than 512 data points, the first data points in those series were removed until 512 trials remained.

#### Spectral analysis

Spectral analysis transforms data series from the time domain (e.g., milliseconds) into a frequency domain, through a fast-Fourier transformation. The procedure finds the best-fitting sum of harmonic sine and cosine waves in a data signal, and renders their power (amplitude^2^) at each fitted frequency on log-log scales. The total number of estimated frequencies in the fast-Fourier transform was 256. The statistic of interest is the slope of the spectral portrait, which captures the relation between amplitudes and frequencies of variation in the data signal. Here, we fitted the spectral slope over the 25 % of lowest frequencies (cf. Eke, Hermán, Kocsis, & Kozak, 2002). As shown in Fig. 1, a zero slope indicates a random signal, and a slope of −1 indicates 1/*f* scaling.

#### Standardized dispersion analysis

Standardized dispersion analysis (SDA) investigates the scaling of variability with changes in sample size. That is, variability is measured using the standard deviation (using the population formula, i.e., using *N*, the number of data points, in the calculation, rather than the usual bias corrected *N* – *1*) of means of progressively larger adjacent samples in a time series. That is, the analysis tracks how dispersion in sample means decreases as progressively larger samples of adjacent data points (bins) are aggregated together in a sample mean. As a first step, the standard deviation is computed for the original data series, which contains 512 data points. The second step involves calculating the standard deviation of the 256 means (bins) of each two consecutive measurements (bin size), and so on. We iterated this procedure until only 16 bins were remaining, each of which represents the mean of 16 adjacent samples in the original time series.

**Fig. 3:**
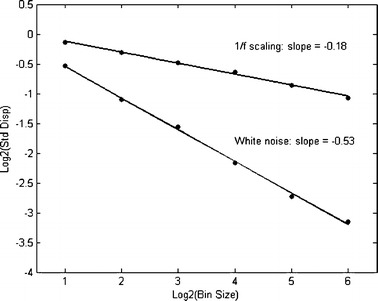
Standardized dispersion is shown as a function of sample-bin size, on log-scales (base 2 was used here). The *solid line* is the least-squares regression for the six different estimates. Fractal dimension is computed as 1—the slope. The fractal dimension of white noise equals 1.5, whereas a fractal dimension of 1.2 indicates exact 1/*f* scaling

The results from SDA can be seen in a plot of the logarithm of the bin size against the logarithm of the standard deviation, as in Fig. 3. For random, white noise, it should not matter that adjacent samples are being grouped and regrouped to form samples of different sizes; for white noise, the slope in Fig. 3 is close to −0.5 (see Van Orden et al., 2003, for a detailed description). The outcome of SDA is expressed by the fractal dimension of a time series, which is given as 1 minus the slope of the regression line. Hence, the FD of white noise is 1.5. A shallower slope (i.e., the 1/*f* scaling pattern shown in Fig. 3 has a slope of around −0.2) indicates correlated activity across timescales, as expressed by the change in a variance statistic due to changes in bin sample sizes. A fractal dimension thus expresses whether the variance statistic converges fast enough, as sample size increases, to yield a stable population parameter. If not, then the process that produced the variance is scale free and has no characteristic scale or quantity of variance. An in-depth tutorial of both spectral analysis and SDA can be found in Holden (2005).

#### Detrended fluctuation analysis

**Fig. 4:**
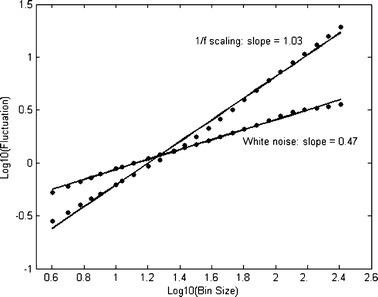
Average fluctuation is depicted as a function of sample-bin size, on log-scales. The solid line is the least-squares regression across timescales. The slope of the regression line equals 0.5 for white noise, and 1 for 1/*f* scaling

Detrended fluctuation analysis (DFA; Peng et al., 1993) is yet another method to reveal the extent of 1/*f* scaling in behavioral time series and is especially useful when confronted with nonstationary signals. The first step is to integrate the time series to be analyzed. Next, the integrated time series is divided into bins of equal length, containing *n* data points. In each bin of length *n*, a least squares line is fit to the data (representing the *trend* in that bin). And then the time series is detrended by substracting the local trend in each bin. From the now integrated and detrended time series, the root-mean-square fluctuation (average fluctuation) is calculated. This computation is repeated over various timescales (bin sizes) to characterize the average fluctuation at each timescale. In the present study, DFA was performed on bin sizes ranging between 2^2^ and 2^8^ data points (ranging from a few seconds to minutes of performance). The results from DFA are usually shown in a plot of bin size against fluctuation, as in Fig. 4, in which the scaling exponent is given by the slope. For 1/*f* scaling, fluctuation will increase with bin size, as indicated by a linear relationship on log scales (yielding a slope of 1). White noise yields a slope of 0.5.

#### A common scale of fractal dimension

The reported fractal dimension statistics were taken from an average of the fractal dimensions across the three estimates (spectral analysis, SDA, and DFA). The outcomes of spectral analyses and DFA were first transformed into a common scale of fractal dimension. For spectral analysis, FD = (*α* ^2^ + 4*α* + 15)/10, where FD is the fractal dimension, and *α* the slope of the power spectrum. For DFA, FD = 0.4*β* ^2^ − 1.2*β* + 2, where *β* is the slope of the log-plot of bin size against fluctuation. A fractal dimension of 1.5 equals white noise, exact 1/*f* scaling yields a fractal dimension of 1.2.

#### Recurrence quantification analysis

RQA combines recurrence plots (Eckmann, Kamphorst, & Ruelle, 1987), that is, the visualization of trajectories in phase space, with the objective quantification of nonlinear system properties. That is, time series are delayed with a certain lag or delay and embedded in a phase space with an appropriate dimensionality (Takens, 1981),

$$ {{X}_n} = \left[ {{{X}_n},\;{{X}_{{n + d}}},\;{{X}_{{n + }}}_{{2d}}, \ldots, \;{{X}_{{n + (m - }}}_{{1)d}}} \right], $$

for each data point *X*_*n*_ in a time series, where *m* is the embedding dimension, and *d* is the delay. The *m* number of variables become coordinates in a geometrical space: the phase space of the system. Next, around each point *X*_*n*_ in phase space, an *m*-dimensional sphere, or radius, is calculated. Every time the time series returns within this radius, after having left it, the points that fall within this radius are called recurrent points.

Consequently, measures like recurrence rate change in function of the a priori choice of radius, and thus the radius needs to be chosen carefully. Setting the radius too large diminishes the sensitivity of the analysis, because many (or in the extreme case all) points would be considered recurrent. Likewise, setting the radius too small would cause very few points to be considered recurrent. In the present study, radius was set to 20 % of the maximum Chebychev distance in phase space, which corresponded roughly to a recurrence rate between 0.05 and 0.10 (cf., Riley et al., 1999) to reassure that the range of individual outcomes was not restricted in either direction for both experimental groups.

Other parameters that affect the outcome of RQA measures, and thus need to be chosen carefully, are the time lag, or delay, and the embedding dimension. Here a delay of 1 data point was combined with an embedding dimension of 6. The choice for delay was based on the average mutual information function (AMI), which is a form of autocorrelation function that provides information about the predictability of *X*(*t* + x) given a measurement *X*(*t*) over a range of possible choices of delay (Fraser & Swinney, 1986). Because it is desirable in a phase space reconstruction for each surrogate dimension to reveal something new about the dynamics (i.e., to reveal the smallest mutual information), the first local minimum of the AMI function was chosen as the optimal delay.

The choice for embedding dimension was based on global False Nearest Neighbor analysis (Kennel, Brown, & Abarbanel, 1992), which reveals how much information would be gained by adding additional surrogate dimensions. That is, when phase space is projected in too small a number of embedding dimensions, non-neighboring points could be misconceived as (false) neighbors. Choosing embedding dimension too high would be not useful either, because there is nothing more to gain by adding another dimension, since the percentage of false nearest neighbors no longer drops while the algorithmic complexity of the analysis increases. A final parameter is the minimal line length for identifying deterministic segments; here, each sequence of minimally 2 recurrent points was considered a recurrent pattern. An additional check to reassure that the outcomes were robust over a range of input parameters was to use different input parameters, which revealed consistent results (cf. Riley et al., 1999; Riley & Van Orden, 2005).

The next step is to quantify complexity measures in the reconstructed phase space. The first measure is recurrence rate, which is computed as the ratio of the number of recurrent points in phase space over the total number of points in phase space. By construction, recurrence rate varies between 0 and 1 (sometimes recurrence rate is displayed as a percentage, however). Determinism is defined as the ratio of the number of recurrent points forming a recurrent pattern over the total number of recurrent points in phase space. Entropy is computed as the Shannon entropy of a histogram in which the number of deterministic segment lengths of different lengths is counted and distributed over integer bins of the histogram, where each bin represents a possible length of a recurrent pattern as empirically determined based on the frequency with which deterministic patterns of different lengths are observed. Entropy is computed as –Σ(*P*_b_)log_2_(*P*_b_), where *P*_b_ indicates bin probabilities of all nonzero bins greater than or equal to the number of recurrent points defining a recurrent pattern (cf. Webber & Zbilut, 1994). For example, if 100 upward diagonal lines—ten each of ten different lengths—are observed, then the probability of a given line falling in a given nonzero bin is 0.1. Meanline is the average duration of deterministic patterns in that distribution. Detailed tutorials that include a careful examination of RQA are Marwan et al. (2007), Riley, Balasubramaniam, and Turvey (1999), and Riley and Van Orden (2005). The RQA analysis was run using Marwan’s CRP Toolbox for Matlab (available at: http://www.recurrence-plot.tk).

## Results

### Group differences

**Fig. 5:**
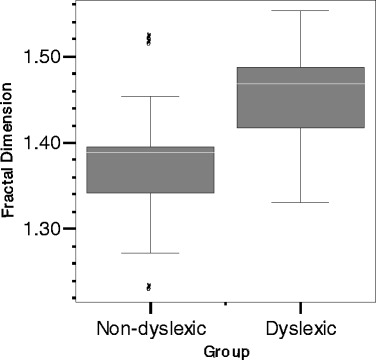
The difference in fractal dimension (*y*-axis) between response series of dyslexic and non-dyslexic readers (*x*-axis)

The descriptive statistics of each of the measured variables are presented in Table 1. As expected, the dyslexic readers showed slower response times, larger intra-individual variability in response time (standard deviation), more pronunciation errors, and lower EMT scores, compared with the non-dyslexic readers. Also the fractal dimension statistic distinguished between dyslexic and non-dyslexic reading performance. As predicted, the temporal structure of response times was closer to white noise in dyslexic response times and clearer examples of 1/*f* scaling emerged in non-dyslexic response times. The magnitude and dispersion of this difference can be seen in Fig. 5.

**Table 1:** Descriptive statistics of the measured variables

	Non-dyslexic readers (*N* = 15)	Dyslexic readers (*N* = 15)	*t* (28)
	Mean (SD)	Mean (SD)	
One-Minute Test	51.80 (9.64)	31.53 (12.35)	5.01**
Response time	704 (152) ms	1,793 (888) ms	−4.68**
Standard deviation	400 (148) ms	1,281 (624) ms	−5.32**
Accuracy	92 (7.6) %	79.2 (10.8) %	3.76**
Fractal dimension	1.38 (0.08)	1.45 (0.07)	−2.62*
Recurrence rate	0.09 (0.05)	0.04 (0.05)	2.21*
Determinism	0.88 (0.04)	0.79 (0.10)	3.16**
Entropy line length	1.88 (0.23)	1.58 (0.35)	2.70*
Meanline	4.01 (0.63)	3.45 (0.68)	2.33*

**p < 0.01; *p < 0.05, two-tailed

**Fig. 6:**
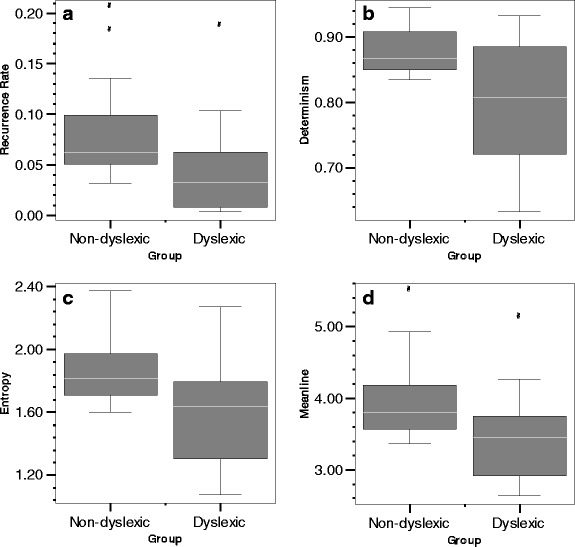
**a**–**d** Differences in RQA measures recurrence rate (**a**), determinism (**b**), entropy (**c**), and meanline (**d**) between dyslexic and non-dyslexic readers

Also, each of the RQA measures differentiated among dyslexic and non-dyslexic readers. Dyslexic response times yield lower recurrence rates compared with non-dyslexic response times (see Fig. 6a). Also, the attractor underlying dyslexic reading performance is less deterministic or patterned, as shown in Fig. 6b, and less complex (Fig. 6c). The final RQA measure, meanline, reveals that the dynamics of dyslexic reading performance are less stable than non-dyslexic reading performance (shown in Fig. 6d).

We may conclude that the temporal dynamics of response variability are sensitive to variations in reading fluency. The word-naming performance of non-dyslexic readers combined fast and stable responses to word stimuli with clearer examples of 1/*f* scaling. Non-dyslexic reading performance also showed a more confined attractor (higher recurrence rate) that is more regular and patterned (higher determinism), more complex (higher entropy), and more stable (longer meanline).

### Within-group correlations among measured variables

With the differences in each of the measured variables between dyslexic and non-dyslexic word-naming performance spelled out, the next step was to investigate the correlations among these variables within each experimental group. Table 2 shows the correlation profile for the dyslexic and the non-dyslexic group separately.

**Table 2:** Two-tailed Pearson correlation coefficients of mean response time (RT) and the One-Minute Test (EMT) with fractal dimension and RQA outcomes for both groups of readers

	Fractal dimension	Recurrence rate	Determinism	Entropy	Meanline
Dyslexic readers					
RT	0.56*	−0.70**	−0.88**	−0.81**	−0.77**
EMT	−0.77**	0.84**	0.75**	0.79**	0.83**
Non-dyslexic readers					
RT	0.21	−0.10	−0.12	−0.25	−0.24
EMT	−0.54*	0.37	0.53*	0.43	0.41

**p < .01; *p < .05, two-tailed

In the dyslexic group, mean response time and EMT reading scores correlate strongly with the fractal dimension statistic and the RQA outcomes. Specifically, more severe cases of dyslexia, indicated by slower response times and lower score on the reading test, produce higher fractal dimensions (less clear examples of 1/*f* noise; range = 1.33–1.55) compared with less severe cases of dyslexia. In addition, a more severe reading impairment is accompanied by a lower recurrence rate, lower determinism, lower entropy, and a shorter meanline. Thus, less severe cases of dyslexia show a higher recurrence rate and more determinism, entropy, and meanline than more severe cases of dyslexia. In the control group, these strong correlations were absent; fractal dimension and RQA outcomes were independent from mean response time and EMT reading scores. The only exceptions are the correlations of fractal dimension and determinism with standardized reading scores, which replicated the relation observed in the dyslexic group in a slightly weakened form.

## Discussion

The present study reveals that young dyslexic readers not just read slower and more variably than non-dyslexic readers. Dyslexic readers equally reveal more random trial-to-trial variability and thus showed less clear examples of 1/*f* scaling in their response times compared with average readers. The results from RQA provided more detailed information about these distinct dynamical patterns of response time. The system dynamics underlying dyslexic reading performance are less confined, patterned, complex, and stable than dynamics underlying non-dyslexic reading performance.

Also the pattern of correlations between aggregate measures of reading fluency (mean response time and standardized reading score) at the one hand, and the dynamical metrics based on 1/*f* scaling and RQA at the other, confirm these differences. While the former metrics loosely relate to the latter metrics in non-dyslexic reading performance at most, the opposite is true for dyslexic reading performance. In the group of dyslexic readers, we observed strong correlations among static and dynamical measures of reading fluency, ranging from 0.5 to 0.9. The implication is that a fuller understanding of learning disabilities like developmental dyslexia may actually require going beyond the aggregate level of analysis of central tendency measures (cf., Gilden & Hancock, 2007).

Together, these findings raise the broader question of the linkage between cognitive dynamics and reading fluency. Why is it that dyslexic naming latencies vary more randomly from trial-to-trial than average naming latencies, and even more so when the reading impairment is more severe? We argue that in cognitive performances, as in physical and physiological systems, the presence of 1/*f* scaling indicates the coupled activity of processes that evolve over multiple timescales. That is, the presence of 1/*f* scaling suggests that the involved components interact so completely that one can no longer parse their individual contributions in the collective activity of the whole system. While each component may contribute its own potentials and constraints, the activity of each component is strongly interdependent with the activities of potentially many other components. This postulate entails that clearer 1/*f* scaling expresses a closer cooperation among task-specific processes nested across different timescales, and could explain why the extent of 1/*f* scaling so strongly relates to functional levels of reading performance (i.e., reaction times and standardized reading scores).

In this study, 1/*f* scaling analyses were assisted by RQA in an effort to understand how well-coordinated behavior (i.e., fluent reading) emerges in the balance of independent versus interdependent component activities. RQA was used to further investigate the emergence of spontaneous temporal order in naming latencies, using the mathematical concepts of self-organization. These concepts inform about how empirically observed temporal patterns can be mapped on simple low-dimensional control principles. That is, in the physical sense, any system described by low-dimensional dynamics is composed of, and coupled to, many subsystems, thereby causing them to fluctuate in an unexpectedly orderly manner over time (i.e., independent trajectories of the system approach each other in phase space). These patterns arise solely as a result of the dynamics of the system with no specific ordering influence from the outside or homunculus from the inside. Therefore, these patterns are referred to as self-organized patterns; the pattern formation is entirely due to the dynamic interaction among the many components that compose the system.

The present results suggest that the cognitive organization under scrutiny is not so much a serial chain of processing components, each adding independently to the duration of each response, but more one of characterizing loops and levels of interdependence in entangled cognitive phenomena (Bell, 1999). But admittedly, one may wonder why one would want to take such a “complex” position to observe reading fluency while “simple” mean values, when contrasted (i.e., in an ANOVA), reveal so much specific information about the system as well. What insights, specifically, are there to gain from this relatively unexplored area of human performance?

Most obviously, we demonstrated that the here introduced methods extend the methodological toolbox available to reading research. The introduced concepts (fractal scaling, recurrence, determinism, entropy, etc.) are mathematically well defined and open to observation with 1/*f* scaling analyses and RQA. More specifically, these methods allow constructing testable predictions for interaction-dominant approaches to human cognition. In the present study, for instance, it was expected a priori that the dynamical properties of word-naming latencies would be so closely related with reading fluency. That is, the more extensive the interdependence among components, the more coordinated and efficient the resultant behavior. Conversely, reduced system interactions, as in developmental dyslexia, yield impaired performance. Although admittedly exploratory, the present study allows for the start of getting a better grip on the full-blown complexity inherent to fluent reading.

The present results allow broader speculation about the nature of developmental dyslexia than before. For instance, in the introduction we raised the question why is it that such a diverse set of empirical findings successfully differentiate between dyslexic and non-dyslexic reading performance. This empirical fact in itself allows that multiple contrasting accounts of developmental dyslexia may be supported simultaneously. The observation, however, that dyslexic children fall out on so many different tasks and modalities is not so strange from the position of interaction-dominant dynamics. Interaction-dominant dynamics do not assume specific component deficiencies to underlie developmental dyslexia, but rather a much more general reduction of system interactions (and hence, coordination) among multiple task-specific processes. It may in fact only be logical that developmental dyslexia shows itself in so many different facets of performance, simply because the linguistic, perceptual, motor, and physiological processes involved in fluent reading so massively interact.

The present findings suggest that background noise and variability more generally may provide a rich soil to further cultivate the dynamic contingencies shared across cognitive activities. Nonetheless, many challenges lay ahead in order to fully understand how far domain-general contingencies of impaired reading performance may reach. For instance, a comparison of timed vs. nontimed measures (cf., Hart, Petrill, & Thompson, 2010) could have allowed for further speculation about the vast comorbidity across cognitive and sensorimotor abilities. While limited in this respect, we nonetheless interpret the present results as a strong motivation to further explore the interactive nature of the embodied (Moreno, Stepp, & Turvey, 2011), genetic (e.g., Petrill et al., 2011), and environmental (Taylor, Roehrig, Hensler, Connor, & Schatschneider, 2010) constraints that may mutually impact word naming fluency.

In sum, the present study reveals that trial-by-trial variability provides psychologists with much more information about the system under scrutiny than would be expected under the assumption of random noise. The finding that the dynamical structure of response time series distinguishes between dyslexic and non-dyslexic readers is new, but aligns with similar findings from other tasks and domains (Diniz et al., 2011; Gilden & Hancock, 2007; Hausdorff, 2007; Goldberger et al., 2002; Kello et al., 2007; Van Orden et al., 2011; Wijnants et al., 2009). In addition, the relative presence of 1/*f* noise and the description of coordination dynamics offered by RQA effectively reveal the severity of the reading impairment. To our knowledge no contemporary models and theories of dyslexia exist that anticipate such an effect, although Greijn (2011) and Van Orden, Holden, Wijnants, & Bosman (2010; based on Holden et al., 2009) might important steps along the way.
